# Coherent behavior of neuromuscular oscillations between isometrically interacting subjects: experimental study utilizing wavelet coherence analysis of mechanomyographic and mechanotendographic signals

**DOI:** 10.1038/s41598-018-33579-5

**Published:** 2018-10-18

**Authors:** Laura V. Schaefer, Frank N. Bittmann

**Affiliations:** Regulative Physiology and Prevention, Department Sports and Health Sciences, University of Potsdam, Potsdam, Germany

## Abstract

Previous research has shown that electrical muscle activity is able to synchronize between muscles of one subject. The ability to synchronize the mechanical muscle oscillations measured by Mechanomyography (MMG) is not described sufficiently. Likewise, the behavior of myofascial oscillations was not considered yet during muscular interaction of two human subjects. The purpose of this study is to investigate the myofascial oscillations intra- and interpersonally. For this the mechanical muscle oscillations of the triceps and the abdominal external oblique muscles were measured by MMG and the triceps tendon was measured by mechanotendography (MTG) during isometric interaction of two subjects (n = 20) performed at 80% of the MVC using their arm extensors. The coherence of MMG/MTG-signals was analyzed with coherence wavelet transform and was compared with randomly matched signal pairs. Each signal pairing shows significant coherent behavior. Averagely, the coherent phases of n = 485 real pairings last over 82 ± 39 % of the total duration time of the isometric interaction. Coherent phases of randomly matched signal pairs take 21 ± 12 % of the total duration time (n = 39). The difference between real vs. randomly matched pairs is significant (U = 113.0, p = 0.000, r = 0.73). The results show that the neuromuscular system seems to be able to synchronize to another neuromuscular system during muscular interaction and generate a coherent behavior of the mechanical muscular oscillations. Potential explanatory approaches are discussed.

## Introduction

Meanwhile, it is a matter of common knowledge that muscles oscillate mechanically in a stochastic manner in a frequency range around 10 Hz^1–6^. This is measured by surface mechanomyography using piezoelectric sensors or accelerometers positioned on the muscle belly^2,5^. It is known that these oscillations characterize the functioning of the neuromuscular system^1^. This is further suggested by the fact that the firing rate of muscles measured by needle electromyography (EMG), inter alia, shows frequencies around 10 Hz^7–12^. A lot of basic research was done concerning the electrophysiological oscillations including intramuscular synchronization effects^7,8,13,14^. Two muscle units (measured by needle EMG) of the first dorsal interossei muscle of the hand and the biceps brachii muscle in healthy subjects are able to show significant coherence in frequency ranges of 1 to 12 Hz and 16 to 32 Hz^15^. Kakuda *et al*.^16^ also found coherence between motor units of the extensor carpi muscle around 12 Hz. Summarizing, motor units of one muscle are able to synchronize in low-frequency areas. In the area of mechanomyography there is not much known concerning the interaction of various muscles of one person or even between the neuromuscular systems of two human subjects. The main focus concerning investigations of the intermuscular interaction deals with intrapersonal coordination in the context of muscle chains^17–22^ or regarding the intermuscular synchronization of electrical activity within one subject. Thereby the firing rate of muscles is measured by EMG. Several researchers report of coherence between EMG signals of at least two different muscles^23,24^. Conway *et al*.^25^ investigated the coherence between EMG of the biceps brachii muscle and the accelerations of the forearm (physiological tremor) with and without motion. During the intervals without motion, the coupling of the signals was apparent especially in the frequency range of 16 to 30 Hz. In motion the coupling occured in two frequency ranges between 8 and 12 Hz as well as between 25 and 40 Hz.

According to previous studies, the synchronization of mechanical oscillations of muscles measured by mechanomyography has not been considered yet – neither intra- nor interpersonal. The purpose of this study is to investigate the mechanical myofascial oscillations of different muscles regarding their coherence during muscular interaction of two persons. This paper is based on the case study reported in Schaefer *et al*.^26^, where already could be shown exemplarly that two neuromuscular systems are basically able to synchronize their myofaszial oscillations in the sense of coherent behavior. The question of the present paper is, whether or not this behavior can be found regularly or rather in single cases. For this, the same measurements were performed with an extended sample. Apart from the interpersonal measurements, the analysis evaluation procedure is novel, too, since a wavelet coherence is used. Wavelet transforms are used in few investigations concerning biomechanics or physiology^26–28^, but are becoming ever more important in such examinations. The use of them can provide benefits, especially for non-stationary data like those of mechanomyographic signals^29^.

## Methods

The aim of this exploratory study is to examine how the mechanical myofascial oscillations behave during isometric interaction between two subjects. Thereby, they are acting against each other with their coupled distal forearms. The MMG and MTG of the triceps muscle and its tendon, respectively, as well as the MMG of the abdominal external oblique muscle are measured and analyzed with algorithms of nonlinear dynamics regarding their coherence.

### Participants

N = 20 healthy subjects (m = 10, f = 10) volunteered to participate in the study and were measured in ten same sex pairs. All subjects were students of the University of Potsdam (studying sports therapy or sport for teaching profession), except for two participants, who were high school students of age 18. The female subjects were aged averaged 21.6 ± 2.1 years, weighed 60.4 ± 3.5 kg, were 168.3 ± 4.4 cm tall and reached an averaged maximal voluntary isometric force (MVC) of 25.85 ± 7.5 Nm. The ten male subjects were averagely 22.1 ± 2.4 years old, weighed 75.2 kg ± 6.9, were 181.5 ± 5.1 cm tall and reached an averaged MVC of 51.19 ± 22.45 Nm. Two subjects were left-handed. The other 18 were right-handed. Exclusion criteria was complaints of the upper extremities, the shoulder girdle and spine within the last six month before the measurement.

**Figure 1:**
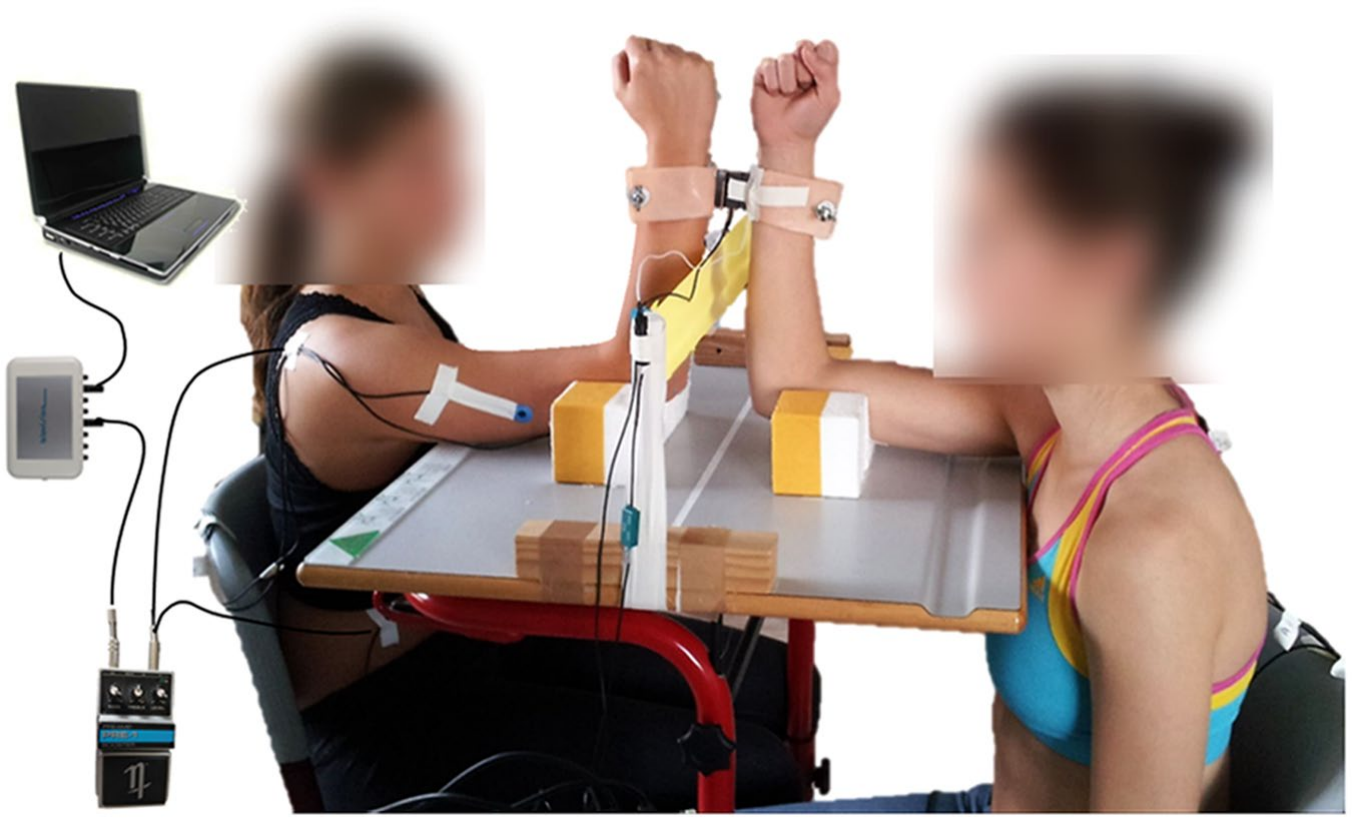
Setting of interacting subjects. Interaction of two subjects during measurement of the abdominal external oblique and triceps brachii muscles and the triceps tendon by piezoelectric MMG-sensors. The signals are conducted across an amplifier to an A/D-converter and are recorded by the software NI DIAdem 10.2 on the measurement notebook (see in^26^).

### Setting

The setting is identically to the case study reported in^26^: The subjects are sitting opposite, but shifted in a way, so that the measured dominant vertically positioned forearms are directly towards each other. The angles between leg and trunk, arm and trunk as well as the elbow angle measure 90° (Fig. 1). An interface proximal of the ulnar styloid processes connects the subjects. It consists of two shells of a thermic deformable polymer material, which is commonly used in rehabilitation technology. The shells are shaped according to the contour of forearms. A strain gauge is located between the shells (model: ML MZ 2000 N 36, modified by biovision) in order to record the reaction force between the subjects. One axis of a biaxial acceleration sensor with a sensitivity of 312 mV/g (range ± 2 g, linearity: ± 0.2%; comp.: biovision) was utilized. The sensor is fixed on the strain gauge to detect the accelerations along the longitudinal acting force vector. The muscle oscillations of the lateral head of the triceps brachii muscle (MMGtri) and its tendon (MTGtri) as well as the ipsilateral abdominal external oblique muscle (MMGobl) are recorded using piezoelectric MMG-sensors (model: Shadow SH 4001). The triceps brachii muscle was chosen since the elbow extension is realized via a single tendon. The abdominal oblique muscle serves as an important stabilizer within the kinematic chain of the measuring position. The MMG-signals are conducted across an amplifier (Nobels preamp booster pre-1) to an A/D-converter (14-bit, National Instruments, modified by Biovision) and subsequently are recorded by the software NI DIAdem 10.2 (National Instruments) on a measurement notebook (Sony Vaio: PCG-61111M, Windows 7). Sampling rate is set at 1000 Hz.

### Measuring procedure

Generally, the subjects should adjust an isometric status at 80% of the weaker subject and maintain this for 15 s (15 s-task). 80% of the MVC was chosen especially because using this intensity the signals of piezoelectric sensors are of very high quality concerning the signal-to-noise-ratio. In a few cases, the couples had difficulties in maintaining the given force level via the interface. Short corrective movements were necessary to readjust the given force. If so, the measurements had to be prolonged for some seconds. Therefore, sometimes the suitable isometric phases were partly shorter or longer than 15 s. During these isometric measurements the subjects of one couple had to perform different tasks: Subject A had to produce actively the isometric force by pushing against subject B, while B had to provide a stable resistance. Results of prior investigations suggest that those two forms of isometric muscle action show different behavior and should therefore be differentiated^30–32^. The authors introduced the terms pushing isometric muscle action (PIMA) for the above mentioned task of A and holding isometric muscle action (HIMA) for the task of B^32^.

In total, eight trials were done: At first, two trials with maximal intensity were performed by each subject seperately by pushing against a stable resistance to determine the individual isometric MVC. The MVC of the weaker subject (highest value of its two trials) was used to calculate the intensities for the further trials. Subsequent, six trials were made for 15 s at 80% of the MVC of the weaker subject. Thereby, three consecutive trials were performed, whereby subject A performs PIMA and B HIMA. For the next three trials the tasks changed. The initial subject to start performing PIMA was randomized. Resting time between the trials was 60 s.

During all trials the subject performing PIMA could control the force level via a biofeedback (dial instrument) over the whole duration time, while the subject performing HIMA should just react to the impacting force of the partner (as a “wall”).

### Data processing and statistical analysis

The isometric plateau of each 15 s-trial at 80% of the MVC was cut from the raw data and was used for the further analysis of frequency and coherence. The criterion for choosing the isometric plateau was the force intensity of 80% of the MVC (±10%). Considerations about HIMA and PIMA are not included in this paper and will be published separately.

The mean frequency of each signal was calculated by a Python script, which inter alia determines the mean frequency out of the average of the intervals between the single maxima. A power spectral density (PSD) was calculated to reflect the frequency distribution as well. More important for the subsequent analysis is the Continuous Wavelet Transform and the Wavelet Coherence performed in Python. The Wavelet analysis enables an investigation of an underlying process on time and frequency^33,34^. The Wavelet coherence is based on the Wavelet transform. It estimates the linear relationship and thereby the coherence of two processes^33,35^. As a function of frequency, it reflects the cross-correlation between two time series.

The Python script was compiled in cooperation with the Department of Applied and Industrial Mathematics, University of Potsdam (Prof. Matthias Holschneider (chair), Hannes Matuschek (assistant)). In this article, the Morlet wavelet was utilized as the mother wavelet^36^. It is defined as *g*_*σ*_(*x*) = $\,{e}^{ix}{e}^{\frac{{x}^{2}}{2{\sigma }^{2}}}$. The procedure used is similar to^37^, except that in the present analysis surrogate data were also estimated.

The Wavelet Coherence enables statements about two non-stationary signals and was utilized to estimate the interaction between both partners and/or – intrapersonally – between different muscles of one partner. It shows the degree of coherence in specific frequency ranges and in the course of time. Thereby, the MMG time-series of the muscles and tendon are related to each other intra- and interpersonally. The contoured patches are significant (α = 0.05; pointwise significance) and are extracted. More detailed information about the wavelet transform see in^26,38^. For further comparisons the time duration of the significant patches was used. Thereby, the four longest significant patches in the frequency range of 5 to 25 Hz were summed up (Sum4Patches). Furthermore, the parameter Sum4Patches was related to the whole isometric duration time (Sum4PaD). Statistical comparisons concerning the parameter Sum4Patch and Sum4PaD were done between randomly selected trials of the real pairs (n = 13) vs. randomly matched trials (n = 13) of each measuring point (in total n = 78), since also the random matched trials show coherence due to analytic specifities. For the group statistics, the data were checked concerning their normal distribution with the Shapiro-Wilk-Test. Further analyses were done using the univariate ANOVA and unpaired t-test for parametric data or the Mann-Whitney-U-test for non-parametric data. Significance level was set at α = 0.05. The effect size was determined either with the Pearsons correlation coefficient r for parmetric data or with the Cohens r for non-parametric data.

### Ethical approval

The study was approved by the ethic committee of the University of Potsdam. It was conducted in accordance with the declaration of Helsinki. All subjects were informed in detail and gave their informed written consent to participate.

### Informed consent

Informed consent was obtained from all individual participants included in the study. Additional informed consent was obtained from the participant for whom identifying information is included in this article (Fig. 1).

## Results

### Torque difference between the interacting partners

The values of MVC recorded in single measurements against a stable resistance amount on average 51.19 ± 22.45 Nm in male and 25.83 ± 7.07 Nm in female subjects. The torque difference of MVC in-between the male couples was on average 15.08 ± 9.40 Nm and in the female couples 3.53 ± 3.19 Nm (range: 0.28…24.77 Nm). With regard to the intensity of 80% of the MVC of the weaker subject during the coupled trials, the stronger partner had an intensity of on average 60.4 ± 11.6% of the MVC in male and 69.6 ± 8.9% in female.

### Frequency

**Figure 2:**
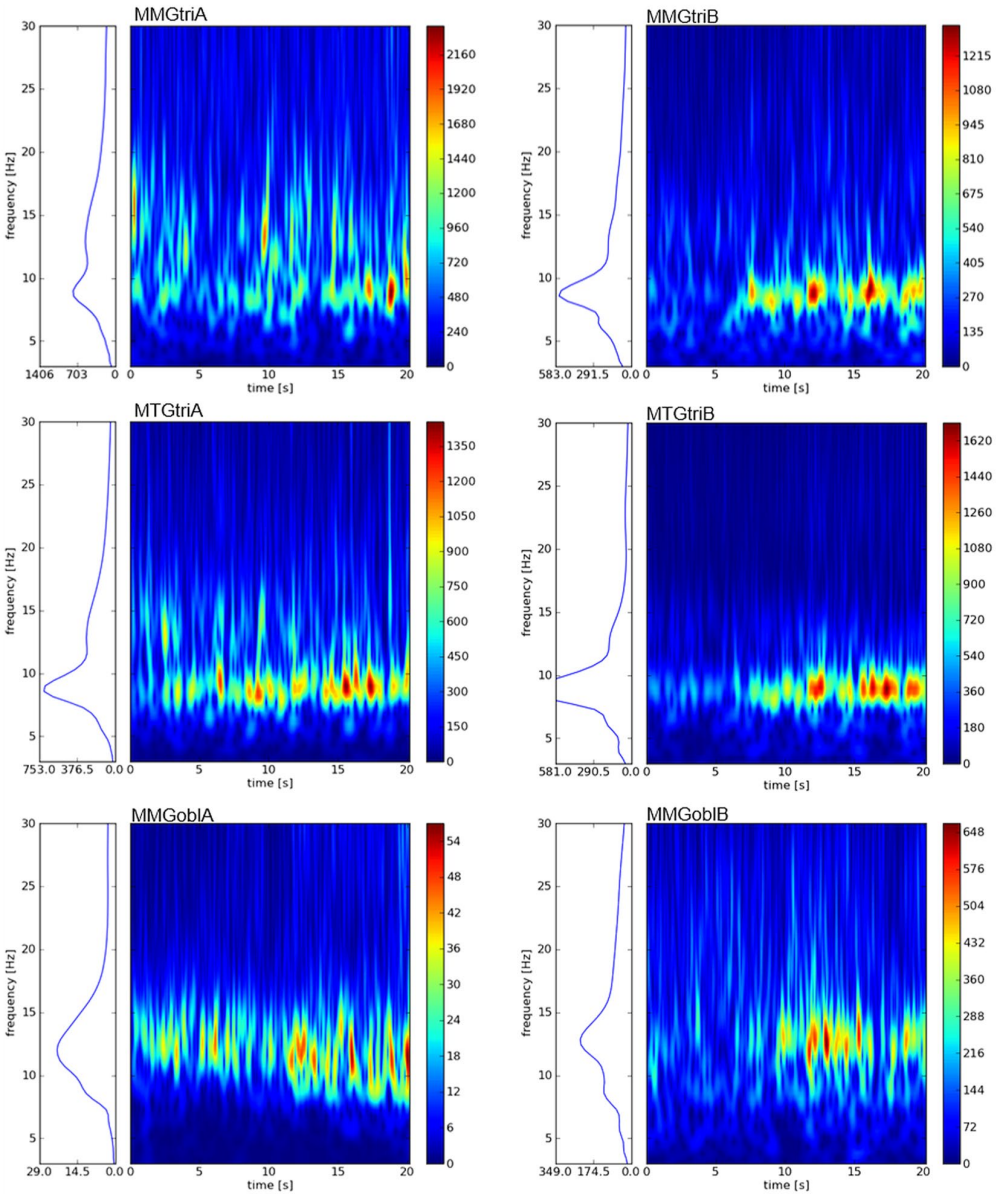
Wavelet spectra. The diagrams show exemplary the wavelet spectra of all three MMG- and MTG-signals of subject (**A**) (left) and subject (**B**) (right) during one trial of isometric interaction at 80% of the MVC. The right scale describes the power. The left panel displays the summation of the power in the wavelet spectrum over time, thus, is equivalent to a smoothed PSD.

Table 1 displays the mean frequency of the whole sample during the 15s-trials. The frequency of MMG-/MTG-signals is to be found in low-frequency ranges between 10 to 18 Hz. The ANOVA shows a significant lower frequency of the MTGtri signals compared with the MMGtri and MMGobl (F(2,108) = 14.822, p = 0.000, η² = 0.215; post hoc test with Bonferroni correction p = 0.000). The MMG-signals of both investigated muscles do not differ significantly (p = 1.0). Figure 2 shows exemplary spectra of the continuous wavelet transform. As can be seen, the frequencies of the single signals are located in low-frequency areas around 8 to 15 Hz, whereas the signals of MMGobl show the highest frequencies.

**Table 1:** Mean frequency of MMG/MTG-signals

	MMGtri	MTGtri	MMGobl
Mean frequency [Hz]	13.85 (±1.39)	12.41 (±1.26)	14.01 (±1.34)

Mean frequency (±SD) [Hz] of MMG/MTG of the triceps muscle (MMGtri) and its tendon (MTGtri) and the abdominal external oblique muscle (MMGobl) signals during isometric interaction at 80% of the MVC of n = 10 pairs (n = 20 subjects).

### Description of the interaction

**Figure 3:**
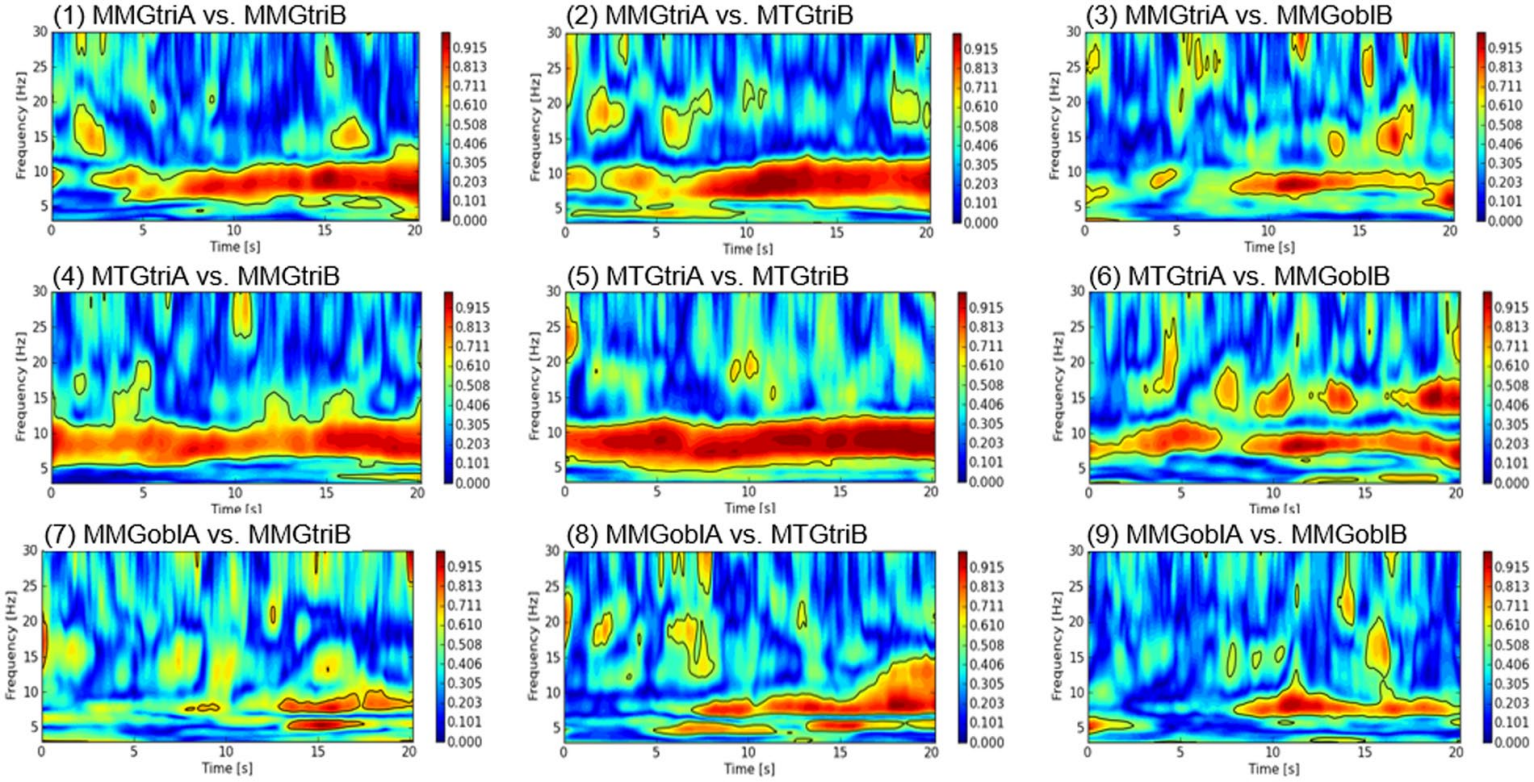
Exemplary wavelet coherence spectra (interpersonally). The diagrams show the wavelet coherence spectra of all three MMG- and MTG-signals of one pair (subject A and B) during one trial of isometric interaction at 80% of the MVC. The intensity of coherence (right scale) in the frequency range of 3 to 30 Hz (y-axis) over the time in s (x-axis) is displayed. The black bordered areas show pointwise significant areas of coherence.

Figure 3 exemplarily shows the wavelet coherence spectra of the interpersonal muscle/tendon pairs of one couple. The black-bordered fields displayed in the wavelet coherence spectra represent significant coherent areas. The right scale of the coherence analysis describes the level of coherence (the redder, the more coherent). Especially in the signal pairs of triceps brachii muscle (MMGtri) and its tendon (MTGtri) of subject A and B a significant coherence over the whole time period is apparent in the range of 6 to 12 Hz (Fig. 3(1),(2),(4),(5)). The signal pairs including the abdominal external oblique muscle (MMGobl) show phases without significant coherence (Fig. 3(3),(6),(7–9). Nevertheless, the significant coherence extends at least over 7.5 s (Fig. 3(7) MMGoblA vs. MMGtriB).

**Figure 4:**
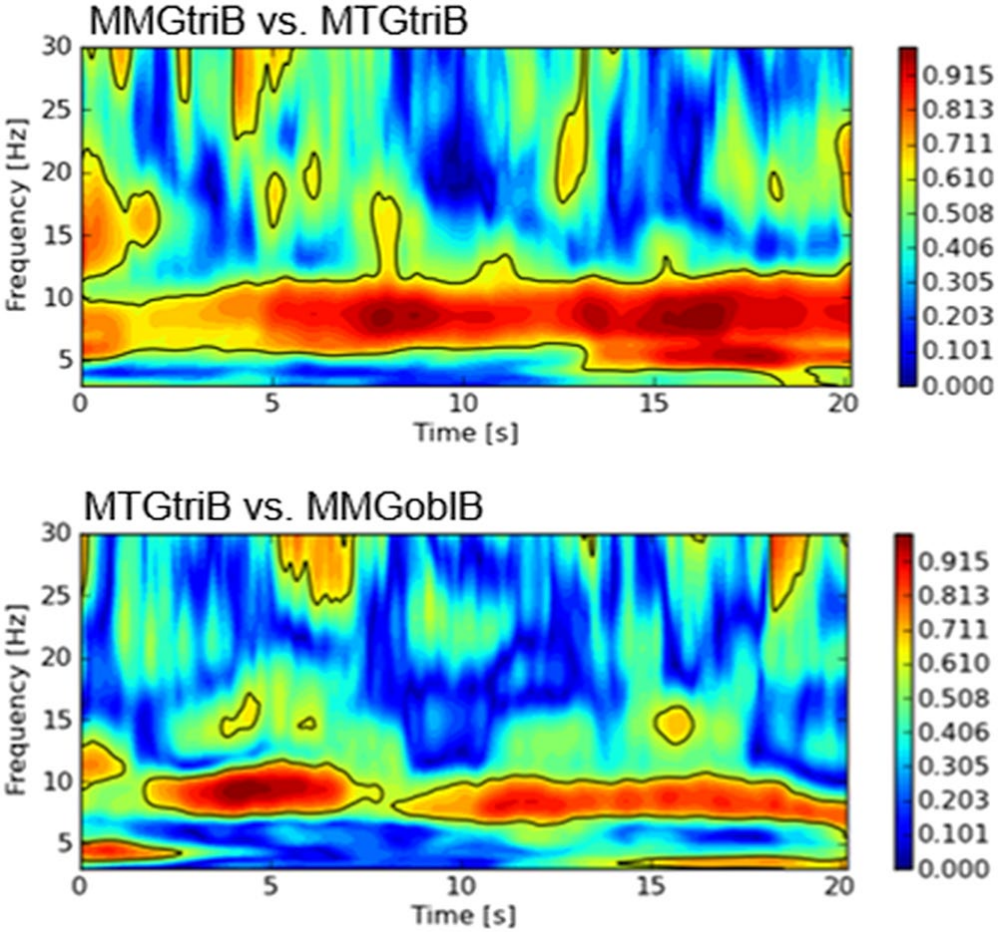
Exemplary wavelet coherence spectra (intrapersonally). The diagrams show the wavelet coherence spectra of the intrapersonal signal pairs of triceps muscle vs. its tendon (above) and of the triceps tendon vs. the abdominal external oblique muscle (bottom) of subject B during isometric interaction of subject A and B at 80% of the MVC.

**Figure 5:**
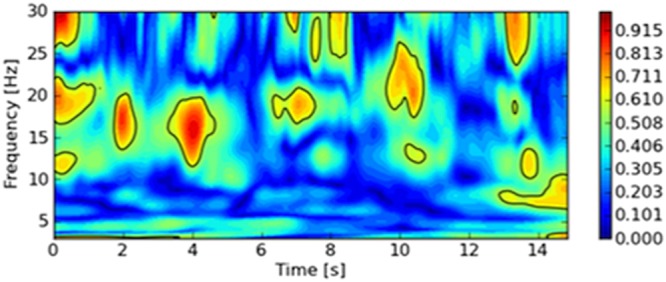
Wavelet coherence spectrum of random matched signals. Exemplary coherence wavelet spectrum of two random matched MMG-signals of the triceps muscle of two different subjects (not paired) of different trials during 80% of the MVC. As the spectrum implies, the durations of the coherence patches are significantly shorter than the real pairs. The patches can be interpreted as spurious coherent behavior.

**Figure 6:**
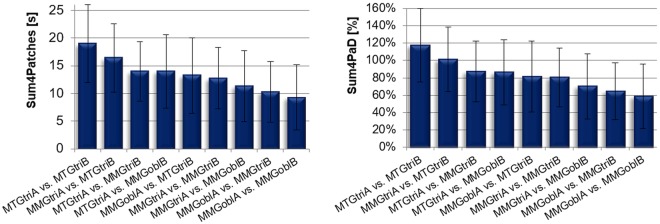
Coherence parameters of interpersonal pairings. The diagrams show the parameters Sum4Patches (left; in s) and Sum4PaD (right; in %) concerning the wavelet coherence analysis (mean and standard deviation) of the interpersonal measurements at 80% of the MVC, differentiated concerning the signal pairs and sized.

**Figure 7:**
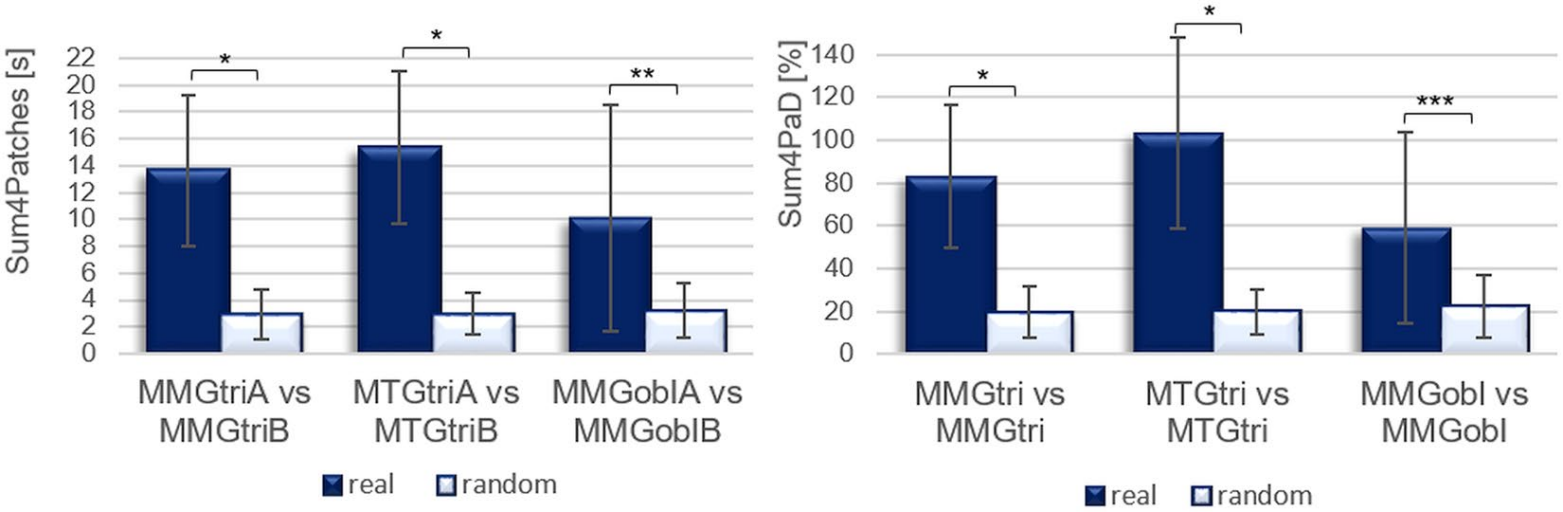
Sum4Patches and Sum4PaD: real vs. randomly matched pairs. The left panel shows the Sum4Patches, the right one the parameter Sum4PaD between the randomly selected n = 13 real pairs and n = 13 randomly matched pairs at each measuring position (mean and standard deviation) of the trials at 80% of the MVC. ^*^p = 0.000 ^**^p = 0.002 ^***^p = 0.003.

As can be imagined, the wavelet coherence spectra of the intrapersonal signal-pairs show an even clearer significant coherence (Fig. 4). Whereas the wavelet coherence spectra of random matched signals display only small and short patches of coherence (Fig. 5).

Figure 6 gives an overview over the arithmetic mean and the standard deviation of the parameters Sum4Patches and Sum4PaD of all signal pairs. The arithmetic mean (M), the standard deviation (SD) and statistical comparisons of those parameters between real pairs (n = 39) and the randomly matched trials (n = 39) are listed in Table 2. Figure 7 visualizes the statistical comparisons. The percentages greater than 100% at parameter Sum4PaD occure because the four longest patches were summed up even if the longest patch already lasts as long as the whole duration time. If patches are overlapping in time, a patch greater than the total duration time can arise. As can be seen, every real signal pairing differs significantly from randomly matched pairs with a high effect size.

**Table 2:** Comparison of parameters Sum4Patches and Sum4PaD between real and random matched signals.

			M (±SD)	t-/U-value	df	p	Pearsons r/Cohens r
Sum4Patches [s]	MMGtri	real	13.658 (5.60)	5.00	—	0.000	0.80
		random	2.927 (1.82)				
	MTGtri	real	15.325 (5.68)	7.541	24	0.000	0.84
		random	2.984 (1.60)				
	MMGobl	real	10.094 (8.44)	23.00	—	0.002	0.62
		random	3.224 (2.09)				
	total (n = 78)	real	13.026 (6.89)	92.00	—	0.000	0.76
		random	3.045 (1.80)				
Sum4PaD [%]	MMGtri	real	0.8306 (0.3353)	6.396	24	0.000	0.79
		random	0.1973 (0.1225)				
	MTGtri	real	1.0335 (0.4476)	6.551	24	0.000	0.80
		random	0.1980 (0.1054)				
	MMGobl	real	0.5902 (0.4450)	28.00	—	0.003	0.57
		random	0.2266 (0.1477)				
	total (n = 78)	real	0.8181 (0.4416)	113.00	—	0.000	0.73
		random	0.2073 (0.1238)				

Displayed are the arithmetic mean (M), the standard deviation (SD) and the results of the group comparisons (unpaired t-test and Mann-Whitney-U-test, respectively) of the real and the randomly matched signal pairs of each measuring point (each n = 13 per signal).

On average, all randomly matched signals (n = 39) show significant coherence patches over 21 ± 12% of the total duration time (Sum4PaD), whereas the significant coherence patches of every n = 485 real signal pairs lasts averagely over 82 ± 39% of the total duration time. The comparison of n = 39 randomly matched vs. n = 39 real pairs (Sum4PaD: 82 ± 44%) shows significant difference concerning the parameters Sum4Patches and Sum4PaD (Sum4Patches: U = 92.000, p = 0.000, r = 0.076; Sum4PaD: U = 113.0, p = 0.000, r = 0.73).

## Discussion

### Torque difference between the interacting partners

The difference between the MVC and therefore the deviation of intensity of partners during the coupled trials, of course, could have influenced the coherence of the oscillations. Especially, the amplitude of the MMG-oscillations depends on the intensity. Up to 80% of the MVC, the amplitude inceases with higher intensity^2^. Therefore, the participant with higher intensity could dominate the amplitude of oscillations. Since in the present consideration of coherence behavior the frequency is the main parameter of interest, it is decisive that the frequency of MMGs are not or only slightly intensity-dependend^2^. Therefore, the difference of intensity and the resulting possible difference in amplitudes should be defer at this point.

### Frequency of MMG and MTG

The results confirm that also during muscular interaction of two neuromuscular systems muscles oscillate mechanically in low frequency ranges of 8 to 15 Hz. This is known for MMG in individual isometric muscle action^1–6^. The frequency characteristics of MMG and MTG-signals detected here are quiet similar. But it is conspicuous that the MTG signals of triceps tendon oscillate with a slightly but significantly lower frequency compared to the MMGs. One could assume that both structures - muscle and tendon - work with the same frequency because they are mechanically coupled. But in contrast to the active force producing muscle, the tendon works as a passive force transitter. Thus, it underlies different mechanical conditions. Furthermore, in this case the tendon has to combine the activities of three muscle heads. We suppose that at the tendon of the triceps brachii muscle a superposition appears, since the oscillations of all three heads are transferred to the tendon. It functions as a kind of passive strand of connective tissue. It seems reasonable that the behavior of oscillations may differ. On the one hand, it would be possible that the frequencies of the other heads are slightly lower than the ones of the measured lateral head. This could possibly influence the tendons’ oscillation. On the other hand, it would be conceivable that tendons could act in the manner of strings, whose oscillations depend on their length but also on the tension, which here is generated by the muscles. The higher the tension produced by the muscle group, the higher the oscillation frequency of the “string”. Thus, possibly the muscular oscillations stimulate the tendon to swing within its own resonance frequency. Nevertheless, the oscillations of the tendon must be expressions of the muscular activity, which was generated during interaction.

### Coherent behavior of muscle oscillations

The results of wavelet coherence analyses of MMG/MTG-signals underpins that the mechanical oscillations of the muscles are coupling. This leads to the conclusion that the neuromuscular systems are able to synchronize between both subjects in the sense of coherent behavior during isometric muscular interaction. As Figure 4 shows, this also seems to be the case intrapersonally. Which explanatory approaches can be considered for this phenomenon? Firstly, it would be possible that the coherence results from a kind of leader-follower-relation, in which one partner drives the other one, who, in turn, follows actively. Secondly, both partners could agree on a different mutual rhythm. According to Pikovsky^39^ partial frequencies are able to generate one interaction frequency during bilateral interaction. Thirdly, it might be conceivable that a kind of master-slave-relation exists, in which only a unilateral coupling is present. The difference with regard to the leader-follower-relation is that the “slave” is not reacting, but is forced into the oscillations of the master. In turn, the “follower” is active, but subordinates its oscillations to the leader ones. Fourthly, the possibility of measurement artifacts has to be taken into account, too. Looking at the last point mentioned: To prevent artifacts, we tried carefully to avoid any mechanical coupling of the sensor cables in the setting as well as other possible external influences by oscillating devices. The PSD does not show any technical harmonic frequencies. Regarding point three, a master-slave-relation would be characterized by asymmetrical levels of activity. In the present interactions, both partners have to be active and generate the common reaction force together. The MMGs show similar oscillating behavior in both partners. Thus, points one and two remain.

We postulate that coherent behavior only can be generated, if both neuromuscular systems are able to adapt to each other. The neuromuscular systems have to adjust to the oscillating partner system. Considering the neurological complexity of intramuscular synchronization, the additional requirements on the sensorimotor systems to organize the myofascial coherence between two subjects must obviously be even higher.

In general, physiological oscillations are able to synchronize to adequate internal or external stimuli^40^. This mostly is referred to the heart rhythm, to cell interactions etc. The authors hypothezise that this also can be transferred to muscular oscillations. In the present setting, it would be conceivable that the pushing partner initiates the external stimuli, to which the holding partner has to react. This would speak for a leader-follower-relation during the coherent interaction, which is able to phasewise form and loose. This theroretical model is further underpinned by the results concerning two different forms of isometric muscle action during isometric interaction^32^, which will be considered elsewhere. A mathematical model of oscillation and interaction is provided in^26^. It seems to be certain that this common rhythm can only be enabled with a kind of clock generator – probably located in the motor cortex^1^.

Since the electrophysiological oscillations of cortex and muscles show coherent behavior intrapersonally in low frequency ranges^41–43^, it is reasonable to assume that also the cortical and mechanical muscle oscillations are able to synchronize within one person. Investigations concerning this topic are in progress. An intrapersonal brain-muscle coherence already has to be based on complex controlling and regulatory processes. Regarding the interpersonal synchronization it is unclear, whether the coherence is generated by spinal or supraspinal controlling processes. Since the sensorimotor systems act as functional units, synchronizations between the motor brain activities are hypothesized here. Indications for this hypothesis are given by several investigations concerning inter-brain synchronization while joint playing music^44–47^ or during other social interactions^48–50^. During coordinated guitar playing^44,46,47^ every participant gets the same acoustical input while hearing the music. The investigators also report that the inter-brain oscillatory couplings were present, when one guitarist was playing and the other was listening. Perhaps the acoustic input already suffices for an inter-brain coupling^46^. During joint playing acoustic guitar, proprioceptive and tactile inputs as well as motor action happen simultaneously. Reasonably, this could trigger mutual EEG patterns. However – in the light of external acoustic impulse generators – the question arises, if there is a real inter-brain coupling. In the present setting an external stimulus like the acoustic one mentioned above does not exist. But possibly, there is a proprioceptive and tactile input from the counterpart. Due to the mutual perception of the joint generated oscillations, we assume that inter-brain synchronization could potentially be present during sensorimotor interaction. A different line of argument could be: EEG/MEG vs. EMG as well as EMG vs. MMG can synchronize intrapersonally during muscular activity^25,41–43,51–53^. If MMGs are able to show coherent behavior interpersonally, it seems to be reasonable that a synchronization of EEG of the involved subjects could appear as well.

### Conclusion and outlook

The present study shows that MMG- and MTG-signals are able to generate coherent behavior interpersonally. Thus, the neuromuscular system is not only able to adjust its own motor action between muscles, but both neuromuscular systems seem to be able to synchronize to each other in the known low frequency ranges of 8–15 Hz. A muscular coupling during motor action must be initiated by the control unit, probably the brain. Nevertheless, the question remains, whether or not an inter-brain synchronization will also be present in EEG. Investigations with EEG and MMG during muscular interaction were done and the evaluation is in progress. Based on the complexity of the neuromuscular control mechanisms, the question arises if disturbances of subjects (e.g. diseases, fatigue or the like) could influence the quality or characteristics of synchronization.

The field of intra- and interpersonal synchronization of parameters of the neuromuscular system could create new insights into the understanding of neurophysiology, pathophysiology and biomechanics of the neuromuscular system. It is still not predictable, which perspectives this will open up in the future.

## Data Availability

The datasets generated during and/or analysed during the current study are available from the corresponding author on reasonable request.
